# Correction: Kawai et al. Effect of Isoflavone on Muscle Atrophy in Ovariectomized Mice. *Nutrients* 2024, *16*, 3295

**DOI:** 10.3390/nu18172769

**Published:** 2026-08-25

**Authors:** Sayaka Kawai, Takuro Okamura, Chihiro Munekawa, Yuka Hasegawa, Ayaka Kobayashi, Hanako Nakajima, Saori Majima, Naoko Nakanishi, Ryoichi Sasano, Masahide Hamaguchi, Michiaki Fukui

**Affiliations:** 1 Department of Endocrinology and Metabolism, Graduate School of Medical Science, Kyoto Prefectural University of Medicine, Kyoto 602-8566, Japan; sayaka25@koto.kpu-m.ac.jp (S.K.); d04sm012@koto.kpu-m.ac.jp (T.O.); c-mori@koto.kpu-m.ac.jp (C.M.); yuka-h@koto.kpu-m.ac.jp (Y.H.); a0911@koto.kpu-m.ac.jp (A.K.); tabahana@koto.kpu-m.ac.jp (H.N.); saori-m@koto.kpu-m.ac.jp (S.M.); naoko-n@koto.kpu-m.ac.jp (N.N.); michiaki@koto.kpu-m.ac.jp (M.F.); 2 AiSTI SCIENCE Co., Ltd., Wakayama 640-0033, Japan; sasano@aisti.co.jp

## Error in Figure

In the original publication [1], there was a mistake in Figure 2 as published. Specifically, the GAPDH panel in Figure 2f was incorrect. In addition, the original raw image corresponding to the published GAPDH blot could not be retrieved. Accordingly, Figure 2 has been replaced with a corrected version, in which panel (f) has been updated using Western blot images obtained from a repeated experiment performed with the same stored frozen samples, together with updated quantification. The authors state that the scientific conclusions are unaffected. This correction was approved by the Academic Editor. The original publication has also been updated.

## Figures and Tables

**Figure 2:**
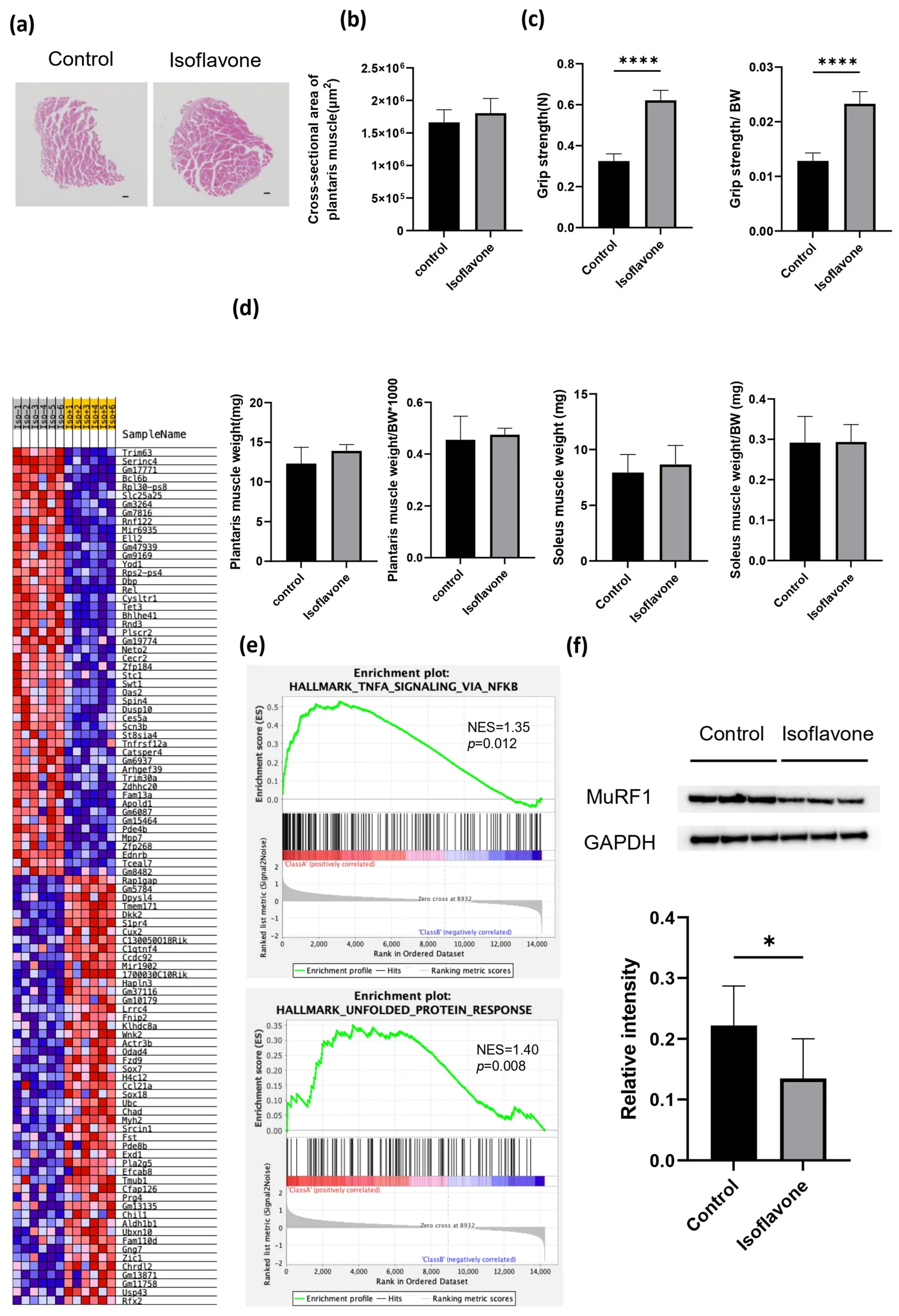
Isoflavone administration enhanced grip strength in mice and decreased the expression levels of muscle atrophy-related genes in OVX mice. (**a**) Shown are representative images of hematoxylin and eosin-stained sections of the plantaris muscle, collected from 14-week-old mice, with a scale bar of 100 µm; (**b**) the cross-sectional area of the plantaris muscle was measured (*n* = 6); (**c**) both absolute and relative grip strength were recorded (*n* = 6); (**d**) the absolute and relative weights of the plantaris and soleus muscles were measured in 14-week-old mice (*n* = 6 in each group); (**e**) heatmap of differentially expressed genes (red, upregulated; blue, downregulated) and gene set enrichment analysis (GSEA) enrichment plots comparing isoflavone-treated mice with controls; (**f**) Western blot analysis of MuRF1 in the gastrocnemius muscle and quantification of the relative intensity of MuRF1 (*n* = 6). Data are presented as mean ± SD values. Data were analyzed using a *t*-test. * *p* < 0.05 and **** *p* < 0.0001.
