# Supplementary figures and images for: Effect of Isoflavone on Muscle Atrophy in Ovariectomized Mice
Source: Nutrients. 2024 Sep 28;16(19):3295. doi: 10.3390/nu16193295 (PMC11478932; doi:10.3390/nu16193295)

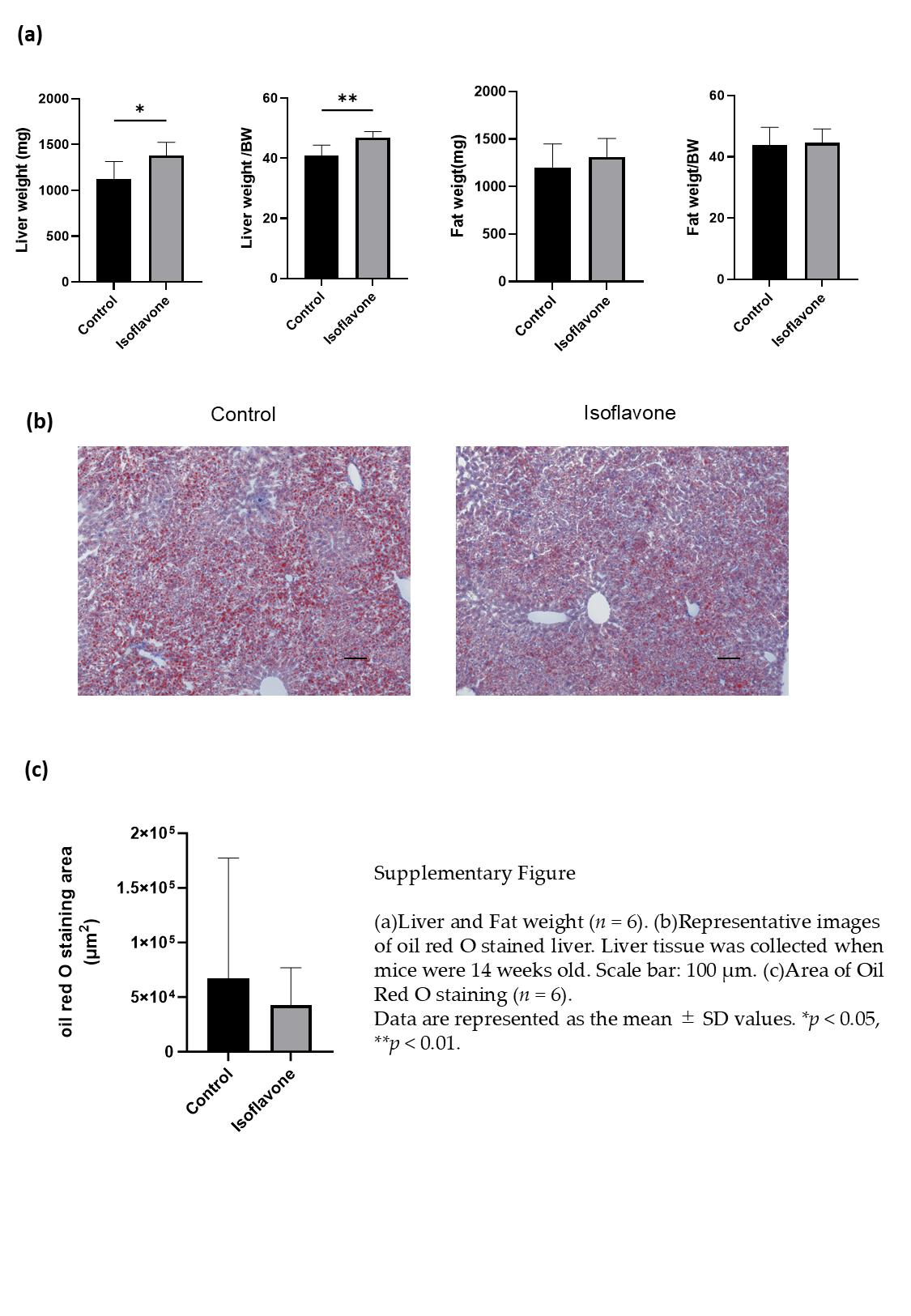

Supplement: Supplementary file 1 [file nutrients-16-03295-s001.zip › Supplementary Figure.png]
